# Double-flow focused liquid injector for efficient serial femtosecond crystallography

**DOI:** 10.1038/srep44628

**Published:** 2017-03-16

**Authors:** Dominik Oberthuer, Juraj Knoška, Max O. Wiedorn, Kenneth R. Beyerlein, David A. Bushnell, Elena G. Kovaleva, Michael Heymann, Lars Gumprecht, Richard A. Kirian, Anton Barty, Valerio Mariani, Aleksandra Tolstikova, Luigi Adriano, Salah Awel, Miriam Barthelmess, Katerina Dörner, P. Lourdu Xavier, Oleksandr Yefanov, Daniel R. James, Garrett Nelson, Dingjie Wang, George Calvey, Yujie Chen, Andrea Schmidt, Michael Szczepek, Stefan Frielingsdorf, Oliver Lenz, Edward Snell, Philip J. Robinson, Božidar Šarler, Grega Belšak, Marjan Maček, Fabian Wilde, Andrew Aquila, Sébastien Boutet, Mengning Liang, Mark S. Hunter, Patrick Scheerer, John D. Lipscomb, Uwe Weierstall, Roger D. Kornberg, John C. H. Spence, Lois Pollack, Henry N. Chapman, Saša Bajt

**Affiliations:** 1 Center for Free-Electron Laser Science, Deutsches Elektronen-Synchrotron DESY, Notkestraße 85, 22607 Hamburg, Germany; 2 Department of Physics, University of Hamburg, Luruper Chaussee 149, 22761 Hamburg, Germany; 3 Department of Structural Biology, Stanford University, School of Medicine, Stanford, California 94305, USA; 4 SSRL, SLAC National Accelerator Laboratory, Menlo Park, California 94025, USA; 5 Department of Physics, Arizona State University, Tempe, AZ, USA; 6 Photon Science, Deutsches Elektronen-Synchrotron DESY, Notkestraße 85, 22607 Hamburg, Germany; 7 Centre for Ultrafast Imaging, Luruper Chaussee 149, 22761 Hamburg, Germany; 8 IMPRS-UFAST, Max-Planck Institute for the Structure and Dynamics of Matter, 22675 Hamburg, Germany; 9 School of Applied and Engineering Physics, Cornell University, Ithaca, New York 14853, USA; 10 Institut für Medizinische Physik und Biophysik, Group Protein X-ray Crystallography and Signal Transduction, Charité - Universitätsmedizin Berlin, Charitéplatz 1, D-10117 Berlin, Germany; 11 Institut für Chemie, Sekr. PC14, Technische Universität Berlin, Straße des 17, Juni 135, 10623 Berlin, Germany; 12 Hauptman-Woodward Medical Research Institute, 700 Ellicott Street, Buffalo, New York 14203, USA; 13 Laboratory for Multiphase Processes, University of Nova Gorica, Vipavska 13, SI-5000 Nova Gorica, Slovenia; 14 Laboratory for Simulation of Materials and Processes, Institute of Metals and Technology, Lepi pot 11, SI-1000 Ljubljana, Slovenia; 15 Helmholtz-Zentrum Geesthacht, Max-Planck-Straße 1, 21502 Geesthacht, Germany; 16 LCLS, SLAC National Accelerator Laboratory, Menlo Park, California 94025, USA; 17 Department of Biochemistry, Molecular Biology & Biophysics, University of Minnesota, Minneapolis, Minnesota 55455, USA

## Abstract

Serial femtosecond crystallography requires reliable and efficient delivery of fresh crystals across the beam of an X-ray free-electron laser over the course of an experiment. We introduce a double-flow focusing nozzle to meet this challenge, with significantly reduced sample consumption, while improving jet stability over previous generations of nozzles. We demonstrate its use to determine the first room-temperature structure of RNA polymerase II at high resolution, revealing new structural details. Moreover, the double flow-focusing nozzles were successfully tested with three other protein samples and the first room temperature structure of an extradiol ring-cleaving dioxygenase was solved by utilizing the improved operation and characteristics of these devices.

Using pulses from X-ray free-electron lasers (XFEL) with sufficiently short duration to outrun radiation damage, the structures of biological macromolecules can be obtained from nanocrystals at room temperature^1^,^2^. After interaction with the pulse, the sample is ultimately vaporized, requiring the recording of many diffraction snapshots, each from a fresh crystal, in order to acquire a complete dataset. The resulting method is referred to as serial femtosecond crystallography (SFX)^2^. New structures^3^,^4^ have been obtained this way, recently even by employing experimental phasing^4^. The approach is well suited for time-resolved crystallography over timescales from femtoseconds to seconds or longer^5^,^6^,^7^, allowing even the investigation of irreversible reactions since each crystal is used only once^7^.

Many SFX experiments to date use a gas-dynamic virtual nozzle (GDVN)^8^ to deliver crystals into the X-ray focus. Such jets can operate in an air or vacuum environment to achieve low diffraction background. Current GDVNs require sample flow rates up to 30–40 μl/min to produce a stable jet of protein crystal suspensions at concentrations in excess of 10^11^ crystals/ml. However, many biological macromolecules of high importance cannot be produced in such large quantities. Using a viscous matrix such as lipidic cubic phase (LCP), sample consumption can be reduced to about 100 nl/min or less^9^. But, the slow flow rates and properties of the viscous jets are not always suitable for many time-resolved light activated measurements due to pre-illumination in a slow jet or for fast mixing experiments due to long diffusion times. The slow flow also impairs their use at high repetition rate X-ray sources such as the European XFEL or LCLS-II. Additionally, the 40–80 μm diameter of the LCP jet leads to higher levels of X-ray background scattering from the carrier medium as compared to typical GDVN jets of around 2–6 μm diameter. Low sample consumption can also be achieved with nanoflow electrospinning injection using a microfluidic electro-kinetic sample holder (MESH), which requires the sample to be mixed into a cryo-protective buffer^10^. An improvement of this method using concentric flow (coMESH)^11^, overcomes this limitation although this injector design is optimized for slow sample flow, rendering mixing experiments at fast time scales difficult.

In the double-flow focusing nozzle (DFFN) that we present here, an inner liquid jet is focused by a coaxial faster outer liquid jet that is itself focused by gas^12^,^13^ as in the traditional GDVN (Fig. 1). This is achieved by injecting the sample stream into the meniscus of the outer sheath liquid where it is being formed into a jet by the gas constriction^12^. In this region, the flow streams are laminar, and the two liquids (sample and sheath liquids) do not have time to fully mix as the meniscus is sped up by the gas flow and drawn out into a fine 2–5 μm jet with the concentrated protein solution at its core (Suppl. Fig. 1). As the jet is primarily formed by the sheath liquid, the sample consumption can be much less than for a GDVN.

## Results and Discussion

**Figure 1:**
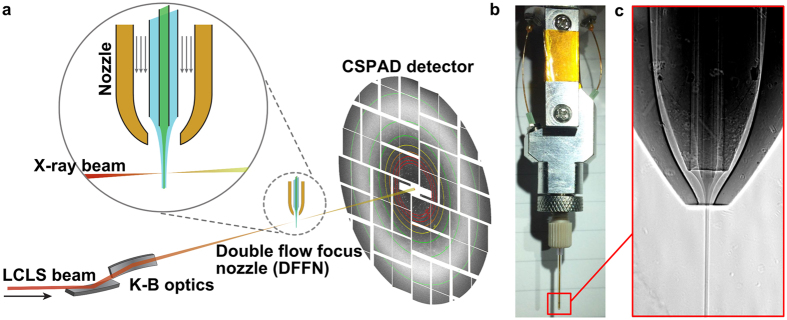
(**a**) Diagram of a SFX experiment at LCLS using a double flow-focusing nozzle (DFFN). Crystals of RNA polymerase II were injected in their crystallization buffer (inner green stream) into the sheath jet formed by ethanol (middle blue stream) through a 40 μm ID capillary. The sheath liquid is accelerated by Helium gas (outer blank channels). A detailed view of the DFFN used in this experiment is shown in (**b**) and in (**c**) a radiograph of a working DFFN, in which the inner jet (4 M KI) can be clearly seen within the sheath jet (ethanol).

**Figure 2:**
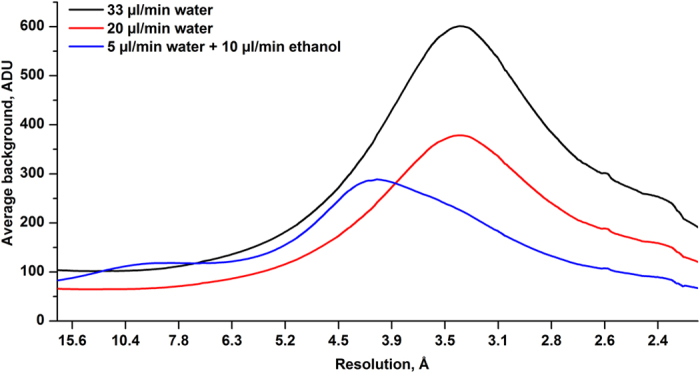
Plots of the diffraction background contribution (in detector ADUs) versus resolution length *d* (Å) for three jet flow rates (black = 33 μl/min water, red = 20 μl/min water, blue = 5 μl/min water plus 10 μl/min ethanol) obtained by averaging the recorded diffraction over annuli of constant scattering angles after first correcting for the linear polarization of the X-ray beam and scaling by pulse energy. DFF injection using an ethanol sheath yields significantly reduced background in comparison to typical water based jets, especially at resolution lengths of 3.5 to 2.2 Å.

**Figure 3:**
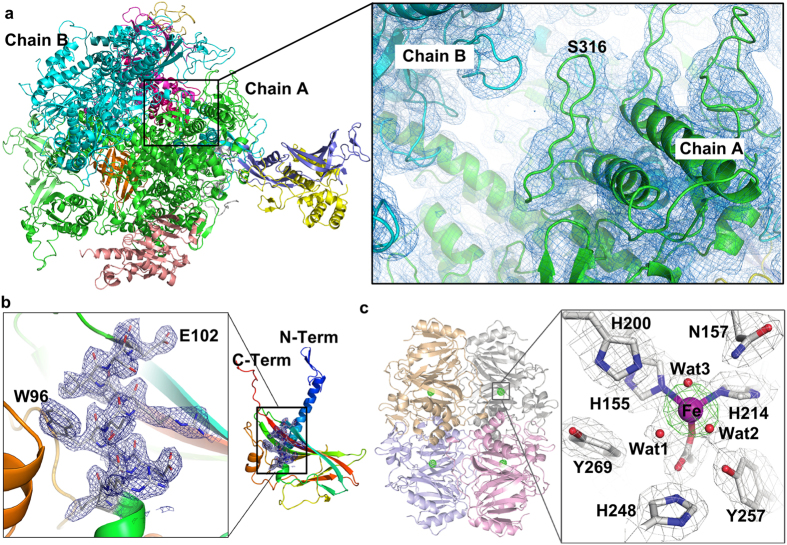
(**a**) Overall room-temperature structure of RNA polymerase II (PDV entry 5U5Q), shown as a cartoon plot. The close-up view shows parts of the interface between chain A and chain B with overlayed 2*F*_obs_ − *F*_calc_ electron density map (contoured at 1.5 σ). (**b**) The asymmetric unit of CpGV (PDB entry 5 MND), with the blue 2*F*_obs_−*F*_calc_ electron density map (contoured at 1 σ) is displayed over an alpha-helical part of the molecule ranging from L92 to E102 and shown as well in the close-up view. (**c**) Crystal structure of HPCD homotetramer (PDB entry 5TRX), and a close-up view of coordination sphere of the catalytic Fe atom in the resting state and key active site residues. The grey 2*F*_obs_ − *F*_calc_ electron density map is contoured at 1.0 σ. The green ligand omit *F*_obs_−*F*_calc_ difference map, contoured at 7.0 σ, was calculated with Fe atoms removed from the model. Atom color code: grey, carbon; blue, nitrogen; red, oxygen; purple, iron. Cartoons depict secondary structure elements for the 4 subunits.

Our DFFN system (Fig. 1) reduces sample consumption up to eight fold compared to a GDVN, can inject crystals in their native crystallization buffers, and is suitable for time-resolved mixing experiments and future high repetition rate X-ray sources such as LCLS-II. Furthermore, low surface tension and low viscosity sheath liquids such as ethanol or n-decane reliably jet at reduced total liquid flow rates and jet diameters, hence further reducing diffraction background (Fig. 2 and Suppl. Figs 2 and 3). Using our DFFN system at LCLS we obtained the first room-temperature structure of RNA polymerase II (RNA pol II, Fig. 3a and Suppl. Table 1). A full dataset, based on 8,854 indexed diffraction patterns (Suppl. Fig. 4), was collected during 160 min of beam time, injecting sub-micron sized crystals (approx. 800 nm width) at an average sample flow-rate of 5 μl/min, consuming 10.8 mg of protein. The SFX structure, refined to 3.8 Å resolution, revealed a number of additional residues previously not observed in the cryo-cooled 12 subunit RNA polymerase^14^ structure (PDB-entry 1WCM), thus providing more complete protein structural information. The majority of these residues are in RPB2 subunit (amino acid residues 70–76, 335–345, 466–477, 717–722), with others in RPB1 (1174–1187, 1243–1254), the RPB4 N-terminus, RPB6 (69–71) and RPB8 (65–67).

Because of the low X-ray background and focusing of the sample, DFFN injection is also well suited for diffraction data collection of very small crystals. *Cydia pomonella* granulovirus (CpGV) crystals are the smallest crystals used so far for structure determination by SFX^15^, with an average size of 400 × 200 × 200 nm^3^ (and about 10,000 unit cells per crystal). The crystal structure of CpGV was determined to 2.56 Å resolution (Fig. 3b) and the statistics (Suppl. Table 1) show that this was limited by the detector acceptance angle rather than the nanocrystal size. Using CpGV, we further analyzed how the fraction of pulses, which show crystal diffraction (the “hit fraction”), depends on the sample flow rate and the type of sheath liquid, for a given crystal concentration. For CpGV, the flow-rate of 3–5 μl/min produced an inner jet width matched both to the size of the X-ray beam and that of the crystals (Suppl. Fig. 5). The average hit fractions of 8–10% obtained at these low sample flow rates indicates the continuous operation of a stable jet over the course of the experiment and the absence of interruptions that often accompany experiments that use GVDNs. Notably, as shown in Suppl. Fig. 5, the hit fraction is higher when ethanol is used as sheath-liquid as compared to water, for the same sample flow-rate. This can be attributed to the lower surface tension of ethanol which leads to a more stable outer jet at lower flow rates, which increases the probability of the center of the jet (where crystals flow) being in the X-ray focus when an X-ray pulse arrives. Even so, either the use of water or ethanol as sheath liquids leads to stable jets at much reduced sample flow-rates as compared to normal GDVN injection. Additionally, we could independently vary the outer and inner flow-rates during operation without impairing the jetting of the DFFN.

**Figure 4:**
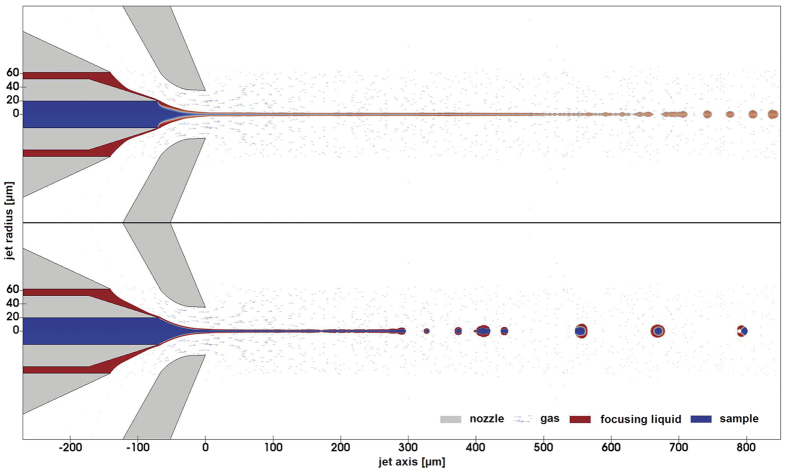
Comparison of a snapshot at the 1 ms time point of two simulations solved with the assumptions of mixing (top) and non-mixing (bottom) of the sample and focusing liquids. Simulations were run in such a way that the focusing liquid - alcohol (red color) jet is created first and is later joined by the liquid of 2.5 M NaCl water solution (blue color), simulating the experimental conditions. The simulation for miscible liquids was obtained with interMixingFoam solver, characterized by a diffusion of 10^−9^ m^2^/s between the miscible phases. The immiscible case shown at the bottom was calculated with multiphaseInterFoam solver. Other parameters were equal in both cases as described in Methods.

We explored a range of focusing liquids and found that the use of n-decane, which is immiscible with water or aqueous buffers, produced a very unstable hit fraction, whereas ethanol and water proved to be good focusing liquids for aqueous suspensions of protein crystals. To better understand the influence of different parameters on the DFFN jet, we performed computational fluid dynamics simulations. Using the nozzle geometry, two cases were studied, one including diffusion between the buffer and the focusing fluid (two miscible liquids) and the other simulating immiscible fluids. The results (Fig. 4) match the experimental observation that sheath liquids miscible with the buffer are better suited for DFFN injection.

DFFN’s are also ideal for jetting samples in buffers that contain high concentrations of polymers (e.g. PEG) and/or salt. With GDVNs operated in vacuum, even low concentrations of PEGs or salts result in an increased risk of ice formation in the X-ray interaction region, which strongly diffracts and can damage the detector. Often only venting of the vacuum chamber removes the ice, reducing the data collection time. The ethanol sheath liquid prevented this ice formation in all tested PEG concentrations (5–15%, w/v). In all tests, the sheath liquid also prevented the formation of PEG and/or salt debris at the nozzle tip, which is very often observed with normal GDVN and can impair data collection in a similar manner to icing.

Previously, injection of the crystal suspension of membrane-bound [NiFe] hydrogenase from *Ralstonia eutropha (Re*MBH)^16^,^17^ with a standard GDVN failed due to the high viscosity of the sample, while a modified GDVN with a 100 μm inner diameter clogged after less than four minutes at flow rates of 30 μl/min. Injection of *Re*MBH using DFFN allowed uninterrupted nozzle flow with a flow-rate of 8.5 μl/min over the course of a 90 minute experiment, enabling the collection of 3576 indexable images. Data collection statistics show that a complete dataset could be obtained to a resolution limit of at least 2.2 Å (Suppl. Table 1, Suppl. Fig. 6). Structural refinement is still on-going and will be published in a separate paper focused on the structure and function of *Re*MBH.

To further test sample delivery with DFFN, micro-crystals of an extradiol ring-cleaving dioxygenase from *Brevibacterium fuscum* (HPCD), at a sample density of only 10^8^–10^9^ crystals/ml, were used to acquire a complete dataset, based on 2,185 indexed patterns, during 49 min of data collection at an average sample flow-rate of 7 μl/min. The SFX structure of the HPCD enzyme in the resting state was refined to 2.38 Å resolution (Fig. 3c, Suppl. Table 1). Comparison of the SFX and cryo-MX (cryo-macromolecular crystallography) structures shows no significant differences in the global protein backbone, but contractions of both macromolecule and unit cell are observed in the low-temperature structure (Suppl. Fig. 7). These changes are absorbed via slight increases in inter-molecular contact areas (Suppl. Fig. 8). Previous cryo-MX studies have shown that the HPCD homo-tetramer remains catalytically active in crystalline form, and that different reaction intermediates accumulate in the 4 nominally identical subunits within a single asymmetric unit^18^. Results of the current study demonstrate that unique crystal packing environments for the four subunits of the homo-tetramer are present at room temperature, supporting earlier conclusions that the observed differential subunit reactivity is due to differences in crystal packing interactions and dynamic restrictions. This finding eliminates cryo-cooling as the cause of differential intermediate stabilization^18^,^19^.

To conclude, a new injection system for SFX experiments, referred to as a double-flow focus nozzle (DFFN), enables structural biology experiments at room temperature on samples of high biological importance as demonstrated by the first structures of RNA polymerase II and HPCD determined at non-cryogenic temperatures. The DFFN significantly reduces sample consumption, overcomes limitations regarding buffer composition and viscosity and substantially extends uninterrupted run-times, while maintaining the benefits of an overall fast flowing jet. Furthermore, it is possible to transform the DFFN into a mix-and-inject nozzle^13^,^20^ for time-resolved structural enzymology experiments (Suppl. Fig. 9).

## Methods

### Double Flow-Focusing Nozzles

A fused silica capillary with a square-profile inner bore of 100 μm width (Polymicro, USA) was used as the outer liquid line. Into this, a 40 μm inner diameter (ID)/105 μm outer diameter (OD) round fused silica capillary (Polymicro, USA) was inserted after slightly etching the inner wall of the outer capillary with hydrofluoric acid to accommodate the 105 μm diameter. All fused silica capillaries were sharpened by wet mechanical polishing^21^ before assembly. Two Kapton rings (so called ‘spacers’) were placed around the outer liquid line (as reported before ref. 21) to ensure centering of this line within the outermost nozzle piece. The inner round capillary self-centers in the square bore of the outer capillary and does not need to be kept in place by spacers. This capillary ensemble was built into the DFFN-housing (see Fig. 1 and Suppl. Fig. 10). A glass capillary with 500 μm ID was flame-polished at its end to form a gas orifice of 60 μm. This outer nozzle piece was then fitted over the outer liquid line and sealed with a fitting-ferrule-sleeve assembly (IDEX). The DFFN was designed so that it would connect the outermost glass capillary to the gas line and the outer liquid capillary to the focusing liquid line. The inner (sample) line extends from the nozzle tip to the upper part of the nozzle housing (~15 cm). The relative position of the tip of the inner liquid line to the tip of the outer liquid line was adjusted after assembly while running liquid in the outer line to optimize nozzle performance.

### Sheath liquids

Focusing liquids were loaded into a stainless steel reservoir and delivered to the DFFN by applying gas pressure on the reservoir. For all experiments, N_2_ was used to pressurize this reservoir. The flow-rate of sheath liquid was adjusted by changing the gas pressure using a Proportion Air GP1 controller. To avoid clogging of the DFFN, a stainless steel in-line HPLC filter (IDEX) with 0.5 μm pore size was placed between the sheath-liquid reservoir and the nozzle. Water was purified with the Milli-Q Integral water system (EMD Millipore) available in the LCLS sample preparation laboratory. Pure ethanol (Sigma Aldrich, CHROMASOLV^®^, SIAL-34852) and n-decane (Sigma Aldrich, SIAL-457116) were used as purchased.

### Numerical simulations

To computationally model the GDVN operation we need to consider its geometrical and surface design, the gas and liquid flow rates (Q_g_ and Q_l_) and pressures (P_g_ and P_l_), as well as material properties of the flow: densities ρ_g_ and ρ_l_, viscosities μ_g_ and μ_l_, and surface tension between the liquid and the gas γ_lg_. The DFFN operation is even more complex, requiring more parameters, since the gas and the sample liquid flow rates and pressures are accompanied by the flow focusing fluid flow rate Q_f_ and pressure P_f_ with accompanying material properties ρ_f_, and μ_f_. The surface tensions between the sample and the focusing liquids γ_lf_, and the focusing liquid and the gas γ_fg_ have to be considered as well, along with the diffusion between the buffer and the flow focusing liquid D_lf_. The adjustment of the DFFN is thus a highly complex task that depends, in addition to the geometrical design, on 16 parameters. The flow rates and pressures of the sample liquid, focusing fluid and gas are thus difficult to optimize in a measurement without prior extensive experimentation. A complementary methodology that helps this optimization of the operating parameters is computational fluid dynamics (CFD). In the case of DFFN this is particularly difficult because compressible gas, two miscible or immiscible fluids, and moving interphase boundaries between all three must be simulated. Compounding the difficulty are the large aspect ratio of the jet length to width and the flow morphology. The flow behavior can be reasonably well represented in axial symmetry only for dripping and jetting modes, whereas the whipping mode^21^ has a highly three-dimensional character.

The OpenFOAM open source CFD solvers “multiphaseInterFoam” and “interMixingFoam” were used respectively for the non-mixing and mixing cases of the sample and focusing liquids. The code for the mixing case had to be modified in order to properly describe the interface between the focusing fluid and the gas. The geometry was prepared by FreeCAD. To reduce calculation time the non-axial symmetry of the DFFN (round inner capillary inserted into square outer capillary) was treated as axially symmetric (consisting of a round capillary inserted into round outer capillary) with the same cross-sectional area of the channel. The simulations were visualized using ParaView, with additional code to extract the length, thickness, and velocity of the sample and fluid focusing jets, frequency and size of drops, drop separation length and other relevant parameters. A typical calculation requires discretization into several hundred thousand computational cells with a width that decreases towards the jet axis to a finest width of 0.3 μm. To assess the DFFN jet behavior it is sufficient to simulate the behavior within the first 3 ms. The respective computational time is several days on a modern server with 20 cores. Figure 4 displays the results of calculations of mixing of the sample and focusing liquids and non-mixing, carried out with the following parameters: Q_g_ = 10 mg/min, Q_f_ = 10 μL/min, Q_l_ = 5 μL/min, μ_g_ = 1.9*10^−5^ kg/ms, μ_f_ = 1.12*10^−3^ kg/ms, μ_l_ = 17.49 * 10^−3^ kg/ms, γ_lg_ = 0.0728 N/m, γ_fg_ = 0.0223 N/m, γ_lf_ = 0.0505 N/m, ρ_g_ = 1.13 kg/m^3^, ρ_f_ = 789 kg/m^3^ and ρ_l_ = 882.4 kg/m^3^. In the case of mixing the diffusion between the miscible phases was set at 10^−9^ m^2^/s. Stable jetting is obtained in both cases, but it is seen that the use of miscible liquids produces a longer and thinner jet, which is preferred for SFX experiments, as compared with immiscible liquids.

### Operation of the DFFN at CXI

For experiments at the CXI beamline we designed and built an adapter to mount the DFFN on the rod assembly currently used to hold and position nozzles in the vacuum chamber (see Suppl. Fig. 10). Using this nozzle positioning system, the tip of the DFFN was positioned approximately 100 μm above the X-ray interaction point. To start the jet, first the flow of sheath gas (He) was started with a pre-tested stable value of around 10 mg/min, requiring about 2.07 × 10^6^ Pa. Then, the focusing liquid reservoir was pressurized with N_2_ to about 1.38 × 10^6^ Pa to get a stable jet with a flow rate between 10–15 μl/min. Once a stable jet was produced, water was injected into the meniscus through the inner capillary using a high-pressure liquid chromatography (HPLC) pump. A stainless steel in-line filter (IDEX) with a pore size ranging from 5 to 20 μm (depending on the sample crystal size) was used. An initial flow-rate of 10 μl/min was selected for the inner flow. Once the jet was aligned with the X-ray interaction region the sample injection switch box at CXI was used to pressurize a sample reservoir plunger using the HPLC pump. Initially the flow-rate of sample was set to be 5 μl/min, and increased in 1 μL/min steps to 10 μL/min if no diffraction hits were observed. Once a hit fraction of about 5% was achieved with 5 μl/min, the flowrate could be slightly reduced to maximize the hit fraction to sample consumption ratio, if desired. To switch between samples, the inner line (sample line) was purged with water until no more hits could be detected by the online analysis (OnDA^22^), followed by a switch to the next sample. To turn off a DFFN without risk of icing or back-flow of sample into the nozzle, the inner channel was first purged with water and then depressurized, then the focusing liquid was depressurized and the DFFN removed from the CXI vacuum chamber with sheath gas flowing. All lines were dried with gas before removing the DFFN from the nozzle rod.

### X-ray radiography of the DFFN

Phase-contrast radiographic X-ray images of the DFFN were acquired at the imaging beamline P05 (PETRA III/DESY and Helmholtz-Zentrum Geesthacht)^23^, experimental hutch (EH2) at 5 keV X-ray energy. Images were acquired using a 3.7 s exposure. In order to enhance image quality a background image, taken using the same exposure but with the injector moved out of the field of view, was subtracted from an average of three subsequent images. For the images shown in Fig. 1c and Suppl. Fig. 1, we used a 4 M aqueous solution of potassium iodide as a sample liquid to provide contrast between the inner and outer streams. Injection was carried out with 5 μl/min sample flow, 20 μl/min outer flow rate (ethanol) and 8.27 × 10^5^ Pa focusing gas (Helium) pressure, which matches typical injection conditions for SFX experiments.

### Preparation and crystallization of RNA polymerase II

12 subunit RNA polymerase II (RNA Pol II) was purified from the yeast *Saccharomyces cerevisiae* as described previously^24^. To make crystals suitable for SFX experiments previously published conditions were adapted to growth of crystals <1 μm^3^ (ref. 25). Pol II was concentrated to 24 mg/ml in 5 mM Tris-Cl pH 7.5, 60 mM ammonium sulfate, 100 uM ZnCl_2_, 10 mM DTT using a Via Spin 500 100 K MWCO spin concentrator. 24 mg/ml pol II was mixed 1:1 with the crystallization buffer (21–22% PEG 400, 5% PEG 5%, 100 mM ammonium phosphate pH 6.3, 100 mM NaCl, 1 mM ZnCl2 and 10 mM DTT) and placed in a 200 μl dialysis button (Hampton Research cat# HR3-330). The dialysis button was sealed using 7000 MWCO SnakeSkin Dialysis tubing (Thermo-Fisher cat#68700), placed in a 50 ml Falcon tube with 20 ml of crystallization buffer, and then in a 16.5 °C incubator. Crystals were harvested after 1 week in the 16.5 °C incubator and stored at 4 °C. Crystal size and concentration were measured using a NanoSight (Malvern). The sample used for the experiments had a size distribution between 500—1000 nm with a mean size of ~800 nm and a concentration of 10^11^ particles/ml. Prior to injection the Pol II crystals were filtered through a 10 μm PEEK filter.

### Preparation of CpGV

*Cydia pomonella* granulovirus (CpGV)^26^ is used as an insecticide against the codling moth (*Cydia pomonella*) and is commercially available in various formulations (e.g. Madex Max, Madex Plus, both Andermatt Biocontrol or CYD-X, Certisusa). Here we used Madex HP (Certisusa). Madex HP contains about 10^13^ virus occlusion bodies (OB) per liter and the OBs were purified from the aqueous suspension by applying iterative washing and centrifugation cycles. The pellet was then re-suspended in ultra-pure water at pH7. After 3 h of incubation at room temperature the supernatant, containing the almost pure OBs, was removed from the pellet and subjected to filtration steps through a sequence of stainless steel filters with decreasing pore sizes (20 μm, 10 μm, 5 μm, 2 μm, 0.5 μm; all IDEX). To increase the concentration of CpGV to the desired 10^11^ particles/ml for injection at LCLS, the suspension was subjected to centrifugation at 21,000 g, the supernatant was removed, and the pellet re-suspended. Size distribution and particle concentration was estimated using a Nanosight LM14 instrument (Malvern).

### Preparation and crystallization of membrane-bound [NiFe]-hydrogenase from *Ralstonia eutropha (Re*MBH)

Native *Re*MBH was expressed and purified in the as-isolated (air-oxidised) state as described elsewhere^16^,^17^,^27^. *Re*MBH nano- and microcrystals were grown at 4°–6 °C and pH 5.5–6.5 in the presence of 20–30% PEG3350 and Bis-(2-hydroxy-ethyl)-amino-tris(hydroxylmethyl)-methane following the micro-crystallization setup-protocol as described for PYP^28^. *Re*MBH solution (approx. 10 mg/ml) was mixed with the crystallization buffer in a 1:1 ratio. The mixture was vigorously stirred for 5 minutes at 4 °C and then incubated for 2–3 days to allow crystal growth to a well-defined size of 5–10 μm and a concentration of 10^11^ small crystals/ml. Prior to injection, crystals were subsequently filtered through 20 μm stainless steel filters.

### Preparation and crystallization of HPCD dioxygenase

Recombinant homoprotocatechuate 2,3-dioxygenase from *B. fuscum* (HPCD) was expressed in *E. coli* and purified as described previously^29^,^30^. For injector-based studies, crystallization conditions and procedures that were optimized to produce diffraction quality macro-crystals of 2,3-HPCD enzymes^18^,^31^ were used as the starting point to optimize and scale up production of micro-crystal slurries using batch crystallization. Protein solutions (5–7 mg/ml) were gently mixed in a 1:1 ratio with crystallization solution consisting of 12–14% PEG6000, 0.1–0.14 M calcium chloride, and 0.1 M MES pH 5.8, ensuring that no visible precipitate forms at these concentrations of components (protein, polymer and salt). After a few minutes of equilibration, a 20 μl aliquot of the 1:1 stock mixture (protein and crystallization solution) was seeded with roughly crushed macro-crystals in an Eppendorf tube, and the growth of many needle-like crystals was observed after a few hours of incubation at 20 °C. The growing seed stocks were diluted with a fresh mixture of protein and crystallization solution every 2–4 hours to maintain maximal growth of needle-like crystals and to reach a total of 2–3 ml sample volume. Following a final 12 hour period of growth to achieve saturation, slurries of needle-like crystals were concentrated by centrifugation at 2,000 rpm for ~5 minutes. Partial fragmentation was achieved by adding layers of 0.1 mm and 0.5 mm glass beads on top of the soft crystalline pellet, and additional centrifugation for 2 minutes. The samples were re-suspended by gentle pipetting, separated from the glass beads and passed through a 20 μm stainless steel filter to remove particulates and larger crystalline material prior to loading into the sample reservoir for diffraction studies. Sample density was estimated to be ~10^8^–10^9^ crystals/ml by bright field microscopy with average crystal needle dimensions of (1–2 μm) × (1–2 μm) × (10–30 μm).

### Data collection

SFX experiments with the DFFN were carried out at the Linac Coherent Light Source^32^ (LCLS) experimental station CXI^33^ at the SLAC National Accelerator Laboratory (Menlo Park, CA, USA) during beam times LH96 (Dioxygenase, Pol II and *Cp*GV) and LG68 (*Re*MBH). The LCLS X-ray beam was focused to 90 × 150 nm^2^ FWHM (V × H) with Kirkpatrick-Baez mirrors. A photon energy of 8.0 keV, pulse length of 50 fs and a repetition rate of 120 Hz was used throughout the experiments. The Cornell-SLAC pixel-array detector^34^ (CSPAD) was used for data collection. To improve the quality of data analysis, the geometry of the CSPAD was refined using ‘geoptimiser’^35^ during analysis of the CpGV data sets. The improved geometry was then used for the other data sets collected. The online monitor OnDA^22^ was used during the experiments to assess hit fraction and data quality ‘on the fly’. This rapid feedback was necessary to adjust the data collection strategy during the experiment for optimization of both hit fraction and sample consumption.

### Data analysis and processing

Individual ‘hits’ were identified from the complete set of collected diffraction patterns and converted to HDF5 format using the software Cheetah^36^. Indexing, integration and data reduction was carried out using the program ‘indexamajig’ in CrystFEL^37^ (Version 0.6.1). The resulting stream-files were subjected to post-refinement (scaling) and merged using ‘partialator’. In the case of CpGV the resulting stream-files were detwinned^38^ using ‘ambigator’ as implemented in CrystFEL to resolve the indexing ambiguity, before post-refinement and merging. MTZ-files for crystallographic data-processing were generated from CrystFEL-hkl-files using f2mtz (CCP4^39^). Figures of merit were calculated using ‘compare_hkl’ (R_split_, CC_1/2_, CC*) and ‘check_hkl’ (SNR, multiplicity, completeness), both from CrystFEL.

### Hit fraction and background analysis

In order to analyze how the hit fraction depends on the sample flow-rate and the type of sheath liquid the CpGV diffraction data was collected at several flow-rates with both water and ethanol as sheath liquid for at least 5 minutes at each point, chosen as a balance between obtaining sufficient data statistics for the hit fraction estimation at a single operating condition and a characterization of the nozzle performance over a range of conditions, given the limited beam time. Changing flow rates on the HPLC can require up to a minute to equilibrate while changes of the gas pressure (driving force for jet velocity) are almost instantaneous. The hit fraction was monitored for 5 minutes after equilibrium was reached. While online monitoring at the beamline was carried out using OnDA, the hit fraction analysis was calculated using the output of Cheetah. The sample flow-rate was measured with a flow meter and then averaged over the run. The sheath liquid flow rate was determined by the set value of the HPLC. Plots of the hit fraction are shown in Suppl. Fig. 5.

For the background analysis three runs were selected: one with the optimal DFFN injection conditions with ethanol as a sheath liquid and two with the flow-rates typical for the water-based jets. 20000 non-hit images from each run were averaged after detector corrections. Then, after applying the correct detector geometry, refined with the ‘partialator’ program in CrystFEL and masking bad pixels and shadowed regions on the detector the radially averaged plots were obtained, shown in Fig. 2.

### Structural refinement

#### RNA polymerase II

Refinement of the room temperature 12-Subunit RNA Polymerase II from *Saccharomyces cerevisiae* was performed using PHENIX^40^ with data spanning scattering angles corresponding to resolution lengths of 40–3.8 Å. Due to small differences in the unit cell dimensions between the room temperature data and previous cryo-cooled structure^25^,^41^ the initial starting model (PDB entry 1WCM) was placed in the unit cell using rigid body refinement followed by simulated annealing, XYZ coordinates and individual *B*-factor refinements with optimized X-Ray/ADP (atomic displacement parameter) weights and secondary structure restraints. The resulting 2Fo-Fc electron density map showed additional density that was built using COOT^42^. Final rounds of refinement were performed with the inclusion of TLS parameters (for translation, libration (small movements) and screw-rotation of a group of atoms). TLS groups were chosen based on analysis using the TLSMD web server^43^. For RPB1 and RPB2, 20 TLS groups were defined; for RPB3-11, 10 TLS groups were defined; and for RPB12, 7 TLS groups were defined. The final refined structure had error metrics *R*_work_ = 0.22, *R*_free_ = 0.27, root-mean-square deviation (RMSD) for angles equal to 0.62°, RMSD for bonds equal to 0.003 Å and a final clash-score of 13.28. The Ramachandran statistics for energetically allowed regions for backbone dihedral angles ψ against φ of amino acid residues were 84.6% favored, 11.8% allowed and 3.6% outliers. Notably, the geometry statistics of the final room temperature 12-subunit RNA Polymerase structure were better than for the previous cryo-cooled structure.

#### CpGV

Rigid-body refinement of the native CpGV structure^15^ (PDB-ID: 5G0Z) against the new data using phenix.refine^44^ did not give a solution. Molecular replacement phasing using PhaserMR^45^ in Phenix^40^ found a single solution (LLG = 3571, TFZ = 46.9). The combination of automatic maximum-likelihood refinement with phenix.refine (xyz, real space and isotropic ADP refinement) and manual model building in Coot resulted in a CpGV structure refined to 2.56 Å resolution. The final refined structure had *R*_work_ = 0.154, *R*_free_ = 0.207, RMSD (angles) equal to 0.54°, RMSD (bonds) equal to 0.003 Å and a final Clash-score of 2.50. The Ramachandran statistics were 97.9% favored, 2.1% allowed, and no outliers.

#### HPCD dioxygenase

The coordinates of the full length 2,3-HPCD (PDB 3OJT) were used as an initial model in rigid body refinement followed by cycles of restrained refinement with Refmac5^46^ as part of the CCP4 program suite^39^ and model building using Coot^42^. TLS was used in the final round of restrained refinement, with a single subunit defined as a TLS group. Link restraints to the iron were removed from the refinement to avoid bias in the refined metal-ligand distances. NCS restraints were not used during refinement, and the 4 subunits of the single enzyme molecule present in the asymmetric unit were refined independently. The final refined parameters for the SFX HPCD structure (PDB 5TRX) resulted in *R*_work_ = 0.18, *R*_free_ = 0.23, RMSD (angles) equal to 1.358°, RMSD (bonds) equal to 0.011 Å and a Clash-score of 2.

### Figures

Figures showing diffraction images were generated using either Cheetah or hdfsee as implemented in CrystFEL. PyMol was used to generate figures showing protein structure models and electron density maps.

## Additional Information

**Accession Codes:** Coordinates and structure factors have been submitted to the Protein Data Bank under accession codes 5U5Q (RNA polymerase II), 5MND (CpGV) and 5TRX (HPCD).

**How to cite this article:** Oberthuer, D. *et al*. Double-flow focused liquid injector for efficient serial femtosecond crystallography. *Sci. Rep.* **7**, 44628; doi: 10.1038/srep44628 (2017).

**Publisher's note:** Springer Nature remains neutral with regard to jurisdictional claims in published maps and institutional affiliations.

## Supplementary Material

Supplementary Information
