# Corrigendum: Double-flow focused liquid injector for efficient serial femtosecond crystallography

**DOI:** 10.1038/srep46846

**Published:** 2017-06-21

**Authors:** Dominik Oberthuer, Juraj Knoška, Max O. Wiedorn, Kenneth R. Beyerlein, David A. Bushnell, Elena G. Kovaleva, Michael Heymann, Lars Gumprecht, Richard A. Kirian, Anton Barty, Valerio Mariani, Aleksandra Tolstikova, Luigi Adriano, Salah Awel, Miriam Barthelmess, Katerina Dörner, P. Lourdu Xavier, Oleksandr Yefanov, Daniel R. James, Garrett Nelson, Dingjie Wang, George Calvey, Yujie Chen, Andrea Schmidt, Michael Szczepek, Stefan Frielingsdorf, Oliver Lenz, Edward Snell, Philip J. Robinson, Božidar Šarler, Grega Belšak, Marjan Maček, Fabian Wilde, Andrew Aquila, Sébastien Boutet, Mengning Liang, Mark S. Hunter, Patrick Scheerer, John D. Lipscomb, Uwe Weierstall, Roger D. Kornberg, John C. H. Spence, Lois Pollack, Henry N. Chapman, Saša Bajt

Scientific Reports 7: Article number: 44628; 10.1038/srep44628 published online: 03 16 2017; updated: 06 21 2017.

In this Article, Henry N. Chapman is incorrectly listed as being affiliated with ‘Department of Biochemistry, Molecular Biology & Biophysics, University of Minnesota, Minneapolis, Minnesota 55455, USA’ and an additional affiliation was omitted. The correct affiliations for Henry N. Chapman are listed below:

Center for Free-Electron Laser Science, Deutsches Elektronen-Synchrotron DESY, Notkestraße 85, 22607 Hamburg, Germany

Department of Physics, University of Hamburg, Luruper Chaussee 149, 22761 Hamburg, Germany

Centre for Ultrafast Imaging, Luruper Chaussee 149, 22761 Hamburg, Germany

In addition, this Article contains typographical errors. In the Abstract,

‘Moreover, the double flow-focusing nozzles were successfully tested with three other protein samples and the first room temperature structure of an extradiol ring-cleaving dioxygenase was solved by utilizing the improved operation and characteristics of these devices’.

Should read:

‘Moreover, the double-flow focusing nozzles were successfully tested with three other protein samples and the first room temperature structure of an extradiol ring-cleaving dioxygenase was solved by utilizing the improved operation and characteristics of these devices’.

In the legend of Figure 1,

‘Diagram of a SFX experiment at LCLS using a double flow-focusing nozzle (DFFN)’.

Should read:

‘Diagram of a SFX experiment at LCLS using a double-flow focusing nozzle (DFFN)’.

In the Methods section, the subheading ‘Double Flow-Focusing Nozzles’.

Should read:

‘Double-Flow Focusing Nozzles’.

