# Supplementary material for: Double-flow focused liquid injector for efficient serial femtosecond crystallography
Source: Sci Rep. 2017 Mar 16;7:44628. doi: 10.1038/srep44628 (PMC5353652; doi:10.1038/srep44628)
Supplement: Supplementary Information [file srep44628-s1.pdf]

Double-flow focused liquid injector for efficient serial femtosecond crystallography - Supplementary information

Dominik Oberthuer[1], Juraj Knoška[1,2], Max O. Wiedorn[1,2], Kenneth R. Beyerlein[1], David A. Bushnell[3], Elena G. Kovaleva[4], Michael Heymann[1,#], Lars Gumprecht[1], Richard A. Kirian[5], Anton Barty[1], Valerio Mariani[1], Aleksandra Tolstikova[1,2], Luigi Adriano[6], Salah Awel[1,2], Miriam Barthelmess[1], Katerina Dörner[1,§], P. Lourdu Xavier[1,2,7], Oleksandr Yefanov[1], Daniel R. James[5,+], Garrett Nelson[5], Dingjie Wang[5], George Calvey[8], Yujie Chen[8], Andrea Schmidt[9], Michael Szczepek[9], Stefan Frielingsdorf[10], Oliver Lenz[10], Edward Snell[11], Philip J. Robinson[3], Božidar Šarler[12,13], Grega Belšak[13], Marjan Maček[13], Fabian Wilde[14], Andrew Aquila[15], Sébastien Boutet[15], Mengning Liang[15], Mark S. Hunter[15], Patrick Scheerer[9], John D. Lipscomb[16], Uwe Weierstall[5], Roger D. Kornberg[3], John C.H. Spence[5], Lois Pollack[8], Henry N. Chapman[1,2,17], Saša Bajt[6*]

* Corresponding author: sasa.bajt@desy.de
1 Center for Free-Electron Laser Science, Deutsches Elektronen-Synchrotron DESY, Notkestraße 85, 22607 Hamburg, Germany
2 Department of Physics, University of Hamburg, Luruper Chaussee 149, 22761 Hamburg, Germany
3 Department of Structural Biology, Stanford University, School of Medicine, Stanford, California 94305, USA
4 SSRL, SLAC National Accelerator Laboratory, Menlo Park, California 94025, USA
5 Department of Physics, Arizona State University, Tempe, AZ, USA
6 Photon Science, Deutsches Elektronen-Synchrotron DESY, Notkestraße 85, 22607 Hamburg, Germany
7 IMPRS-UFAST, Max-Planck Institute for the Structure and Dynamics of Matter, 22675 Hamburg, Germany
8 School of Applied and Engineering Physics, Cornell University, Ithaca, New York 14853, USA
9 Institut für Medizinische Physik und Biophysik, Group Protein X-ray Crystallography and Signal Transduction, Charité - Universitätsmedizin Berlin, Charitéplatz 1, D-10117 Berlin, Germany
10 Institut für Chemie, Sekr. PC14, Technische Universität Berlin, Straße des 17. Juni 135, 10623 Berlin, Germany
11 Hauptman-Woodward Medical Research Institute, 700 Ellicott Street, Buffalo, New York 14203, USA
12 Laboratory for Multiphase Processes, University of Nova Gorica, Vipavska 13, SI-5000 Nova Gorica, Slovenia
13 Laboratory for Simulation of Materials and Processes, Institute of Metals and Technology, Lepi pot 11, SI-1000 Ljubljana, Slovenia
14 Helmholtz-Zentrum Geesthacht, Max-Planck-Straße 1, 21502 Geesthacht, Germany
15 LCLS, SLAC National Accelerator Laboratory, Menlo Park, California 94025, USA
16 Department of Biochemistry, Molecular Biology & Biophysics, University of Minnesota, Minneapolis, Minnesota 55455, USA
17 Centre for Ultrafast Imaging, Luruper Chaussee 149, 22761 Hamburg, Germany
now at Max-Planck-Institute for Biochemistry, Munich, Germany; § now at European XFEL GmbH, Hamburg, Germany; + now at Paul Scherrer Institut, Villingen, Switzerland

Detailed description of the first room temperature HPCD structure and comparison with structures obtained from data collected at cryogenic temperatures

Structure determination using micron-sized crystals of HPCD establishes the feasibility of this enzyme system for time-resolved studies using the serial data collection approach to extend the current *in crystallo* kinetic approaches. Previously, Kovaleva and Lipscomb showed that when a reaction with an aromatic substrate is initiated in HPCD macrocrystals, under limited concentrations of O_2, nominally identical subunits of the homotetrameric enzyme accumulate reaction intermediates at different stages of catalysis (Kovaleva, 2007). The observed differential subunit reactivity *in crystallo* was attributed to the differences in crystal packing environments and associated perturbations in protein dynamics, in the absence of structural differences in global or local conformation among the 4 subunits comprising a single asymmetric unit (Kovaleva, 2007; Kovaleva, 2008). To understand and exploit the role(s) of protein dynamics in enzymatic catalysis, it is crucial to access structural environment and interactions at ambient temperature to eliminate any effects of cryo-cooling used in standard macromolecular crystallography (MX) analysis. Therefore, the SFX structure of the resting state of HPCD determined in this work gives an opportunity to evaluate potential global and local differences in the macromolecular structure determined: 1) by different experimental methods (SFX or MX), 2) varied sample size (single macro-crystal or microcrystalline slurry), and 3) temperature (293 K or 100 K).

Comparison of the HPCD structure determined using the SFX approach with that determined previously at 100K (PDB entry 3OJT, Fielding, Kovaleva, 2011) shows no significant differences in the global protein backbone or changes in the vicinity of the active site. This is evidenced by the RMSD values obtained when superimposing all atoms within each homo-tetramer (0.63 Å), individual subunits (0.53 - 0.58 Å) or those within a 15 Å radius from the catalytic Fe centers (0.38 - 0.46 Å). Furthermore, in both structures, the catalytic Fe atom retains a distorted octahedral geometry coordinated by the so-called 2-His-1-carboxylate catalytic triad, and only minor variability in the average conformations is observed for some key active site residues that participate in catalysis and/or stabilization of reactive intermediates. Specifically, in the SFX structure of HPCD homo-tetramer, insufficient electron density is observed in 2 of the 4 subunits (B and C) for Fe coordination site typically occupied by solvent-derived ligand (Wat1) in the resting state. This may indicate increased mobility at ambient temperature of the solvent-derived ligand that is not stabilized by additional hydrogen-bonding interactions with 2nd coordination sphere residues, in contrast to coordinated solvent molecules Wat2 and Wat3. It is also possible that the lower resolution of the SFX structure (2.38 Å), in contrast to MX structure at 100K (1.70 Å), accounts for diminished electron density observed at the Wat1 site in these subunits. However, minor differences in coordination geometry of the 2-His-1-carboxylate triad (E267 and H214) in subunits refined as hexa- (A and D) and penta-coordinated (B and C) Fe sites would be more consistent with "missing" or more mobile Wat1 ligand rather than the lower resolution

of the dataset per se. Higher resolution of the SFX structure, potentially achievable by measuring many more patterns, is required to resolve these coordination differences.

A common consequence of the cryo-cooling is contraction of both the macromolecule and the macromolecular lattice, driven largely by entropic re-packing. Comparison of the HPCD structures determined at 293K (SFX) and at 100K (MX) shows that the unit cell volume is reduced by 8.2% (Suppl. Figure 8A) and the 4 protein subunit volumes contract by 1.8 – 3.9 % upon cryo-cooling, all without change in the space group. These values fall within the range typical for protein crystals. For the four crystallographic independent subunits of the HPCD homo-tetramer, the cryo-cooling-induced volume contraction coincides primarily with an increase in the number and extent of inter-molecular (crystal packing) and intra-molecular (subunit interface) interactions rather than internal changes which might cause conformational changes within active site architecture (i.e. RMSD for superposition are low). Analysis of accessible surface areas and contacts for the SFX structure at 293K shows that nearly 30% of the accessible surface area for each of the 4 subunits is involved in quaternary (subunit interface) and inter-molecular (crystal packing) interactions. The distribution of the residues involved in crystal contacts differs significantly for each subunit at 293K (Suppl. Figure 8A). Cryo-cooling and its associated lattice re-packing associated with the volume contraction further increase the contact surface area slightly, but preserve the differential distribution of crystal contacts for each of the 4 subunits (Suppl. Figure 8B). Consequently, the unique environments and dynamic restrictions on each subunit of HPCD homo-tetramer imposed by the crystal lattice are in effect during reaction initiation and intermediate accumulation *in crystallo* at room temperature prior to cryo-cooling. This finding supports the attribution of differential subunit reactivity to varying crystal lattice effects as suggested by previous kinetic MX studies at 100K (Kovaleva, 2007; Kovaleva, 2008).

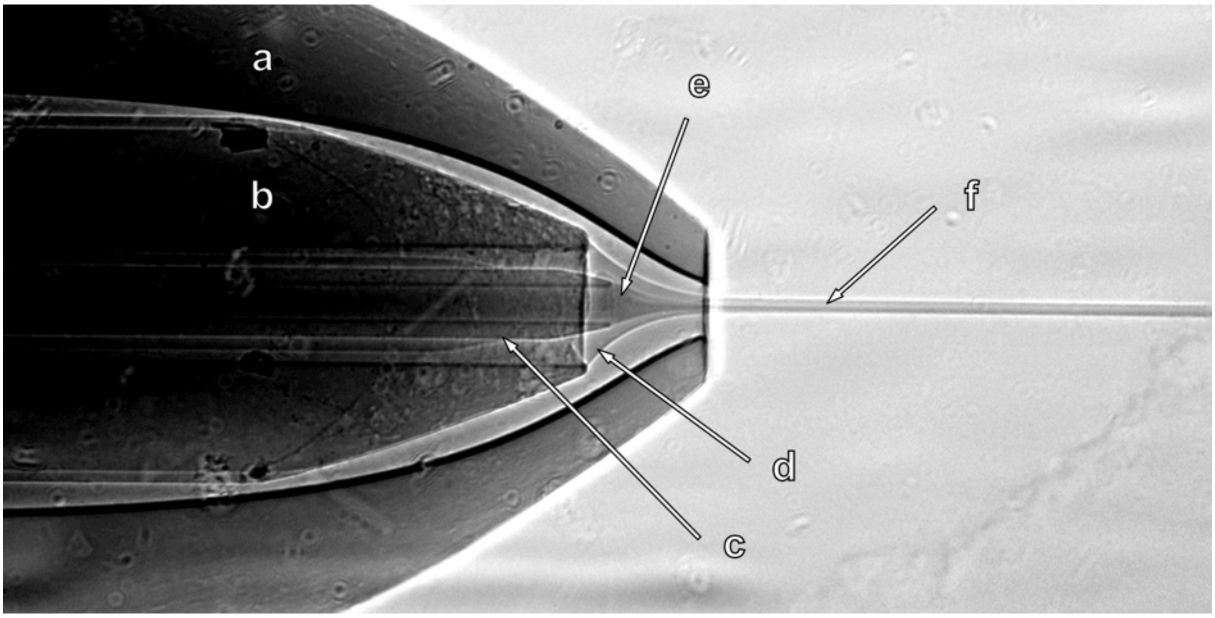

Supplementary Figure 1 X-ray radiograph of a running double flow-focusing nozzle (DFFN). The exposure time was 3.7 s. Here the sample, consisting of a 4 mol/l solution of KI, flowed at 5 µl/min and the outer liquid was ethanol flowing at 20 µl/min. Helium gas at 8.27×10^5 Pa pressure was used to focus the liquids. **a**, Outer glass capillary; **b**, sharpened outer liquid capillary; **c** sharpened inner liquid capillary; **d,** outer liquid (ethanol) stream being focused by focusing gas and forming a jet; **e**, inner jet (sample jet) is focused by the flow of the outer liquid and propagates as a thin jet within the ethanol jet, **f**.

a

b

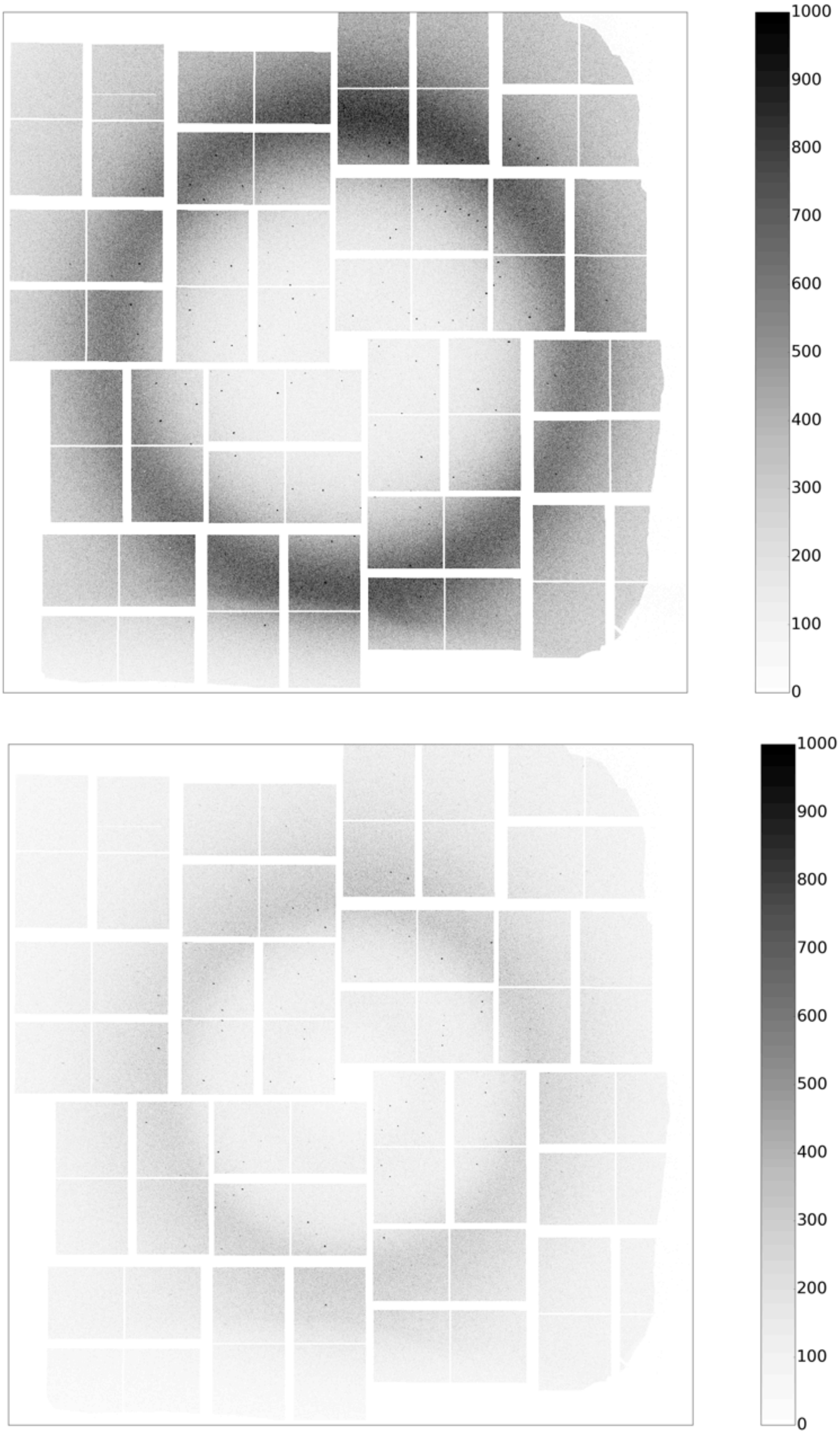

Supplementary Figure 2 a, Diffraction background for a single-pulse exposure of a typical water-based jet flowing at 30 µl/min as compared with **b**, a DFFN jet flowing with a combination of 3 µl/min sample and 10 µl/min ethanol. In both cases a crystal hit was selected and the sample was CpGV. The DFF jet gives less background overall, peaked at a lower scattering angle. The greyscale for both patterns varies from white at 0 counts to black at 1000 adu counts per pixel.

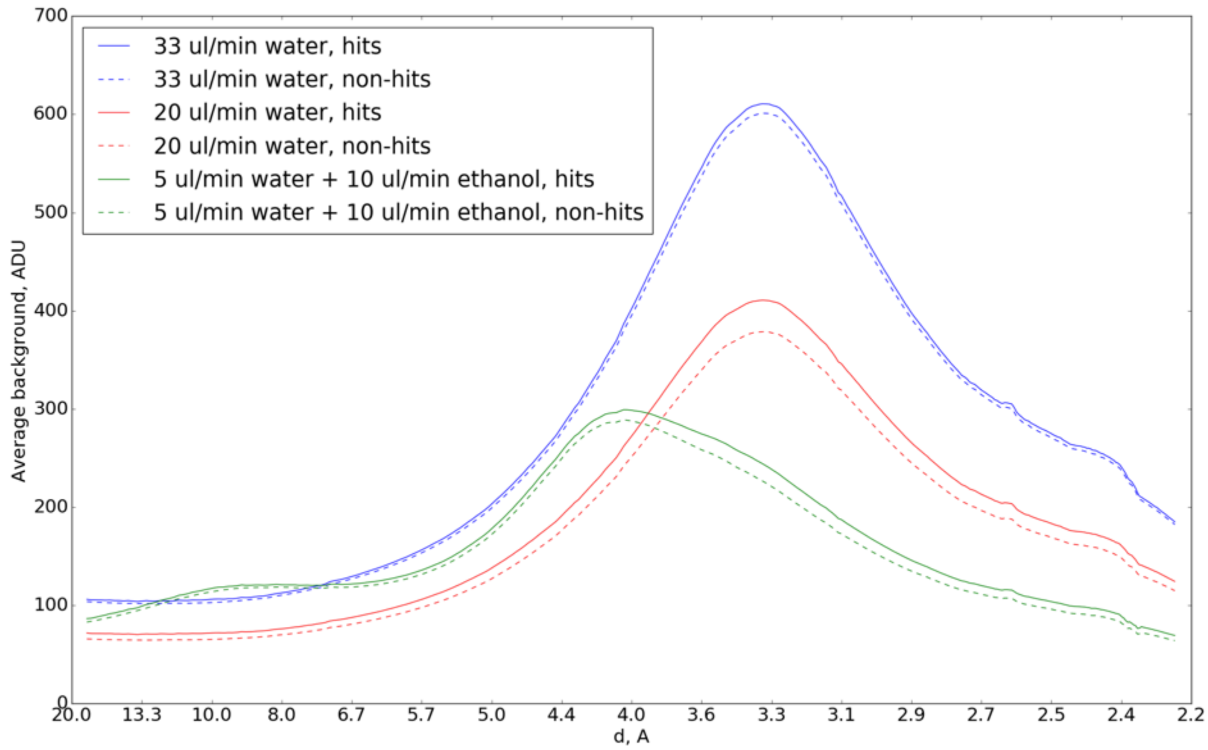

Supplementary Figure 3 Plots of the background contribution (in detector ADUs) versus resolution length *d* obtained by averaging the recorded diffraction over annuli of constant scattering angles after first correcting for the linear polarization of the X-ray beam and scaled by pulse energy. Here a comparison of the averaged 'non-hit'-fraction of patterns (solid lines, as shown in Figure 2) to the 'hit'-fraction (dashed lines) is shown, where the sorting of patterns was achieved using Cheetah. No significant differences between the background contribution of 'hits' and 'non-hits' are apparent. The higher background for the 'hit'-fraction can be attributed to the higher probability of hitting a crystal when the thickest part of the jet is directly in the X-ray focus.

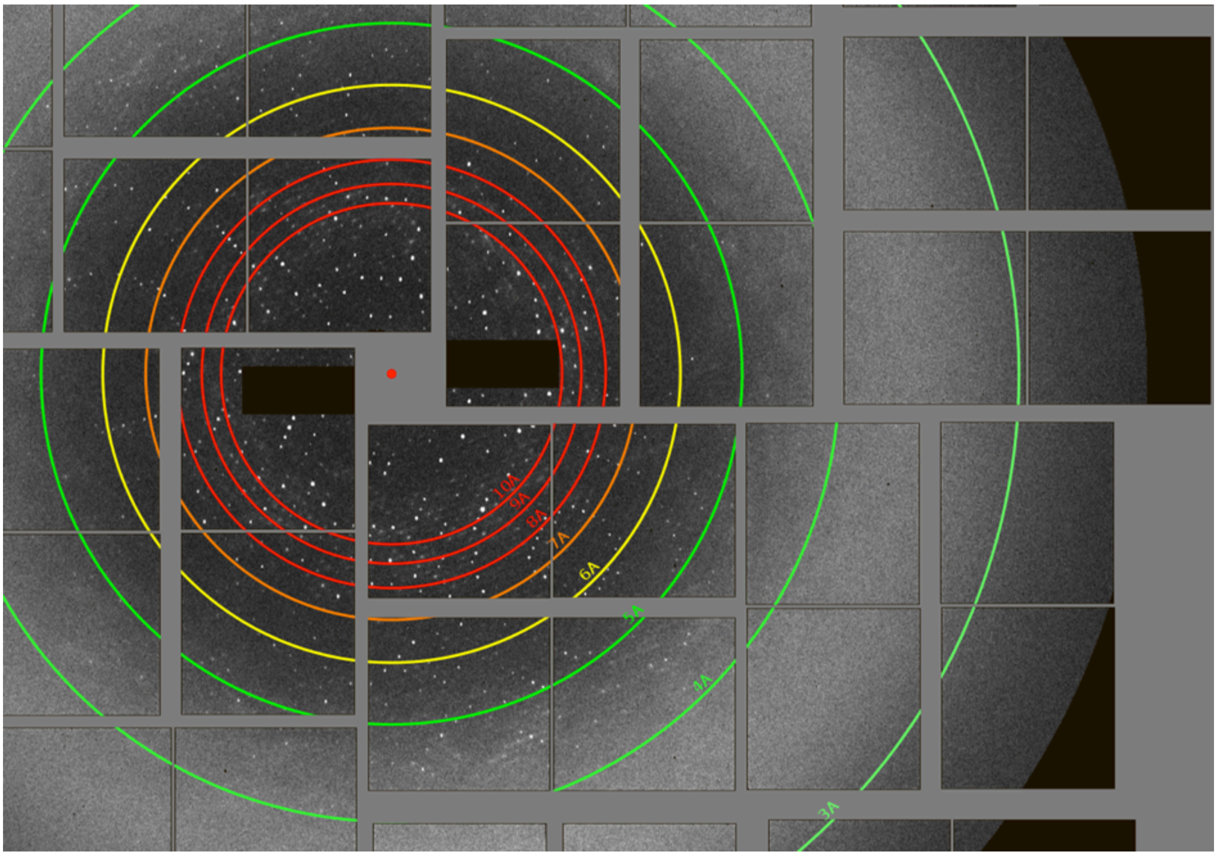

Supplementary Figure 4 RNA polymerase II (Pol II) diffraction pattern obtained at CXI during experiment LH96 using the DFF injector with ethanol as the sheath liquid. Diffraction is visible up to 4 Å in this single-shot frame.

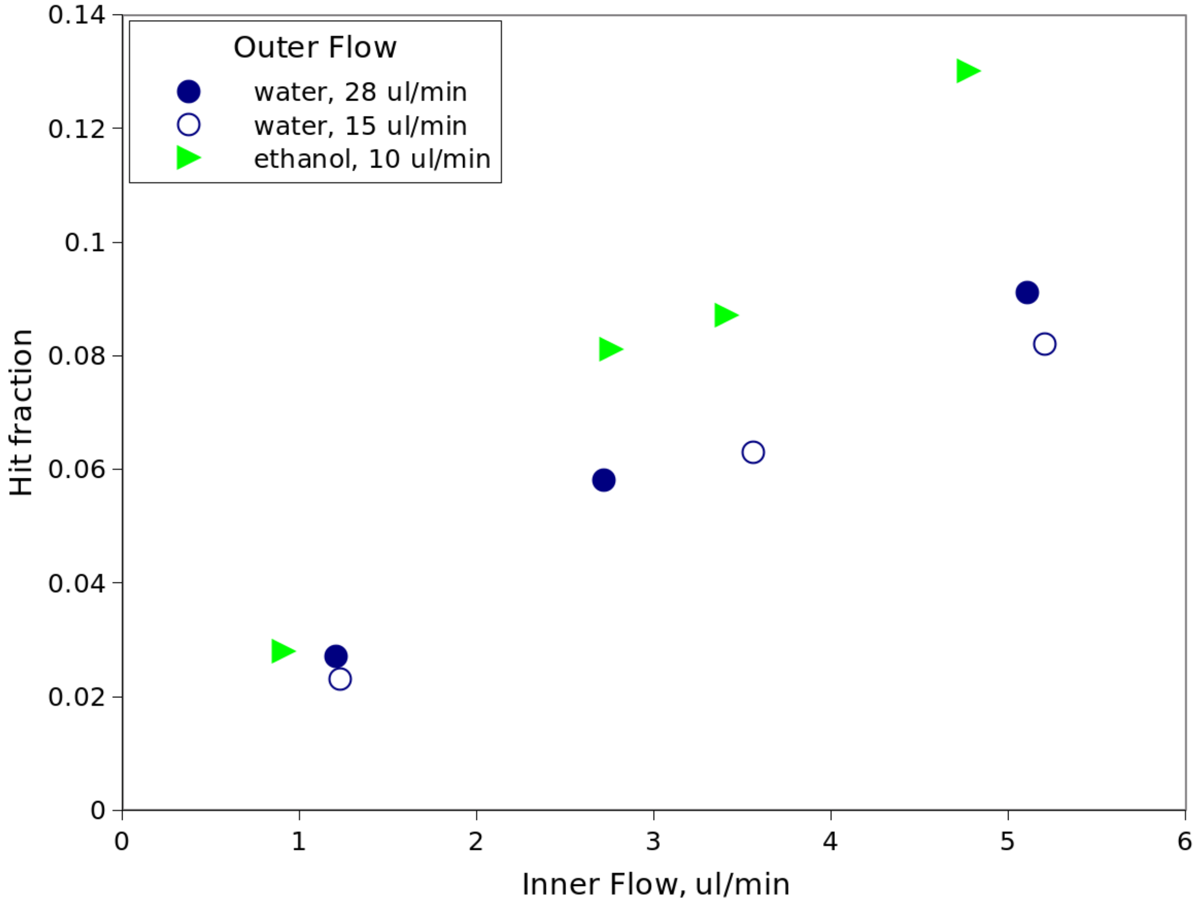

Supplementary Figure 5 Plots of the hit fraction as a function of sample flow rate for water and ethanol as sheath liquids. It is seen that ethanol increases the hit fraction as compared with water at sample flow rates ≥ 3 μl/min.

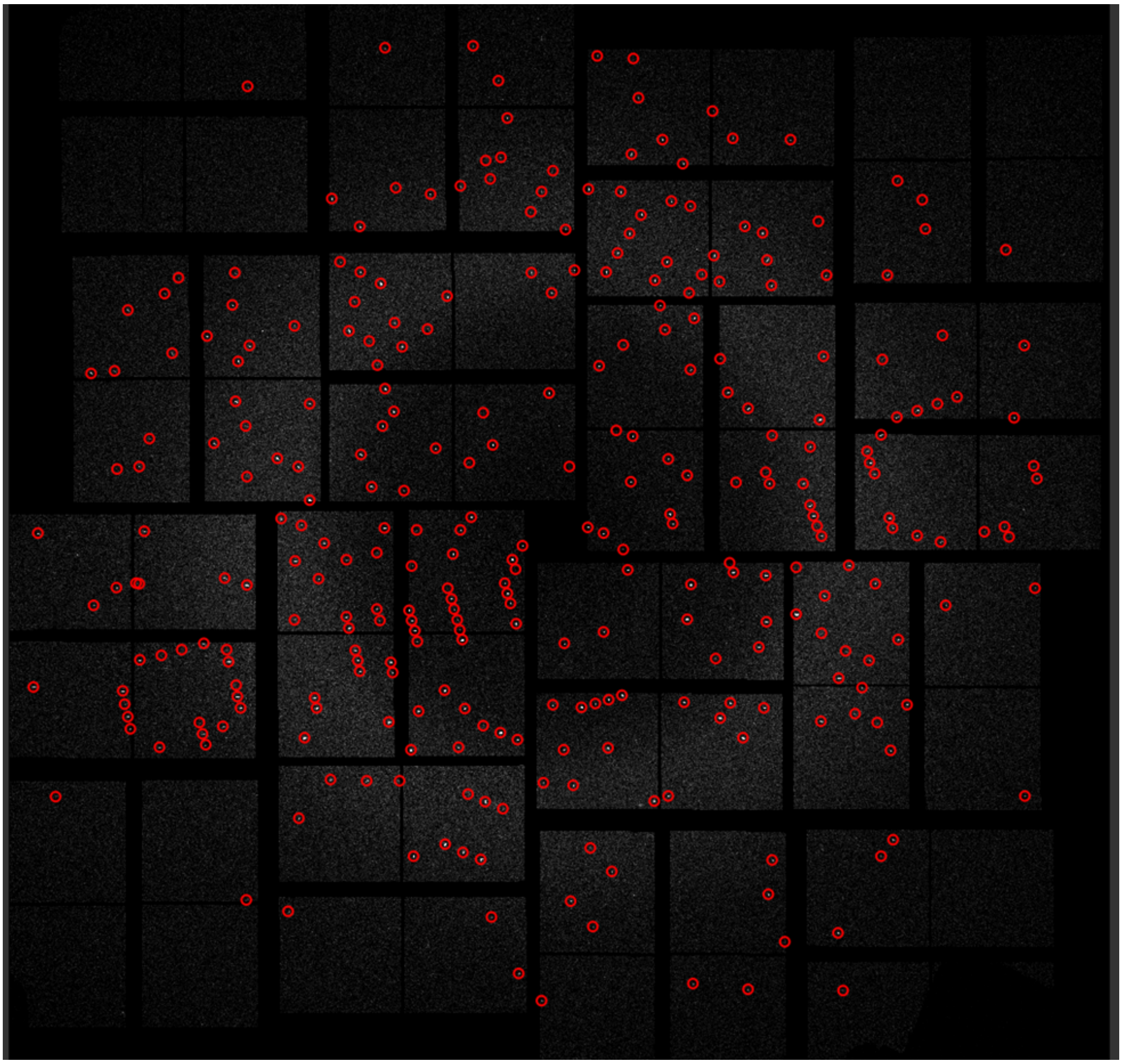

Supplementary Figure 6 Membrane-Bound [NiFe] Hydrogenase (MBH) diffraction pattern obtained at CXI during experiment LH96 using the DFF injector with ethanol as the sheath liquid. Diffraction is visible up to the edge of the detector (2.2 Å, limited by shadowing of the "shroud" used for differential pumping of the jet environment to the main CXI chamber).

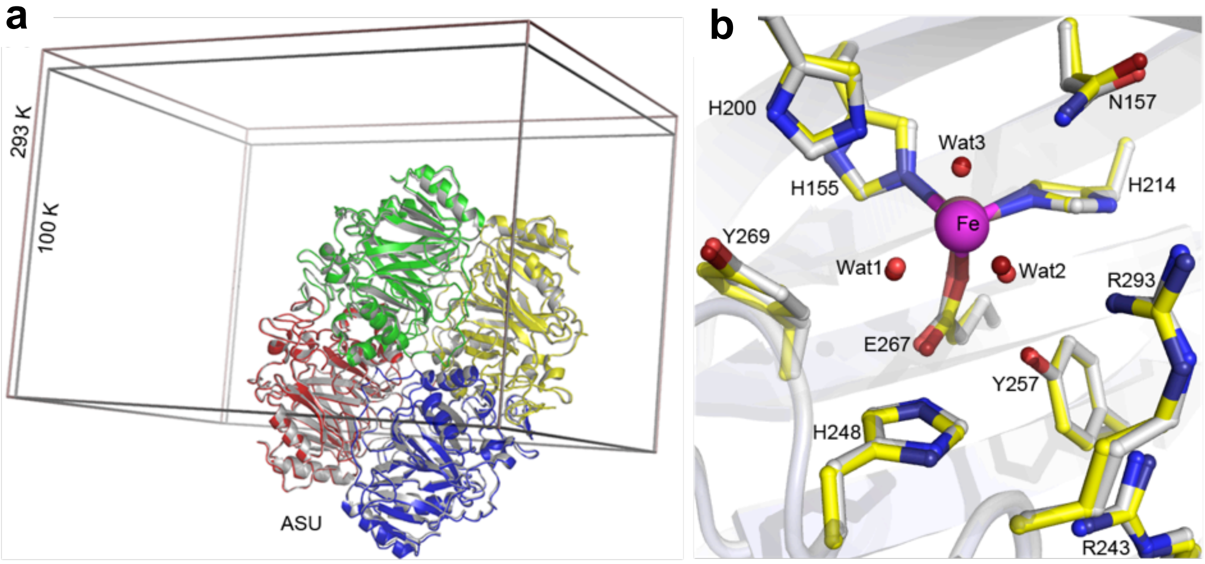

Supplementary Figure 7 Comparison of HPCD structures determined by SFX and cryo-MX methods. **(a)** Superposition of HPCD structures determined at 293K and 100K, illustrating change in unit cell volume. Cartoons depict secondary structure elements. Subunits color code: colored (293K); grey, (100K). **(b)** Overlay of active sites for HPCD structures determined at 293K (PDB entry 5TRX) and 100K (PDB entry 3OJT). Atom color code: yellow, carbon (293K); gray, carbon (100K); dark blue, nitrogen (293K); blue, nitrogen (100K); dark red, oxygen (293K); red, oxygen (100K); purple, iron (293K); bronze, iron (100K). Cartoons depict secondary structure elements.

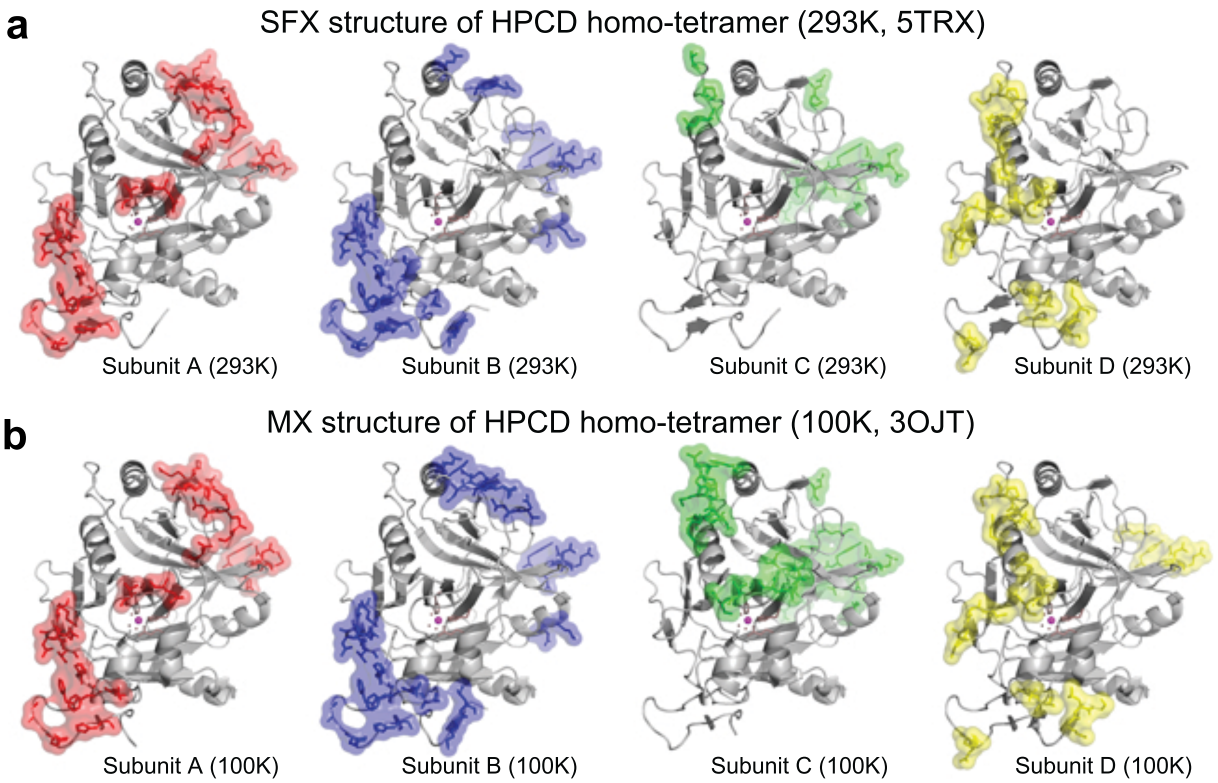

Supplementary Figure 8 Distribution of residues involved in inter-molecular (crystal packing) interactions for subunits of HPCD homo-tetramer before (**a**, 293K, PDB entry 5TRX) and after (**b**, 100K, PDB entry 3OJT) cryo-cooling. Surface residues involved in crystal contacts with symmetry-related molecules are shown as colored sticks and surfaces, illustrating cryo-cooling induced increase in intermolecular contact surface area, but overall preservation of the unique environments for each subunit. Cartoons depict secondary structure elements.

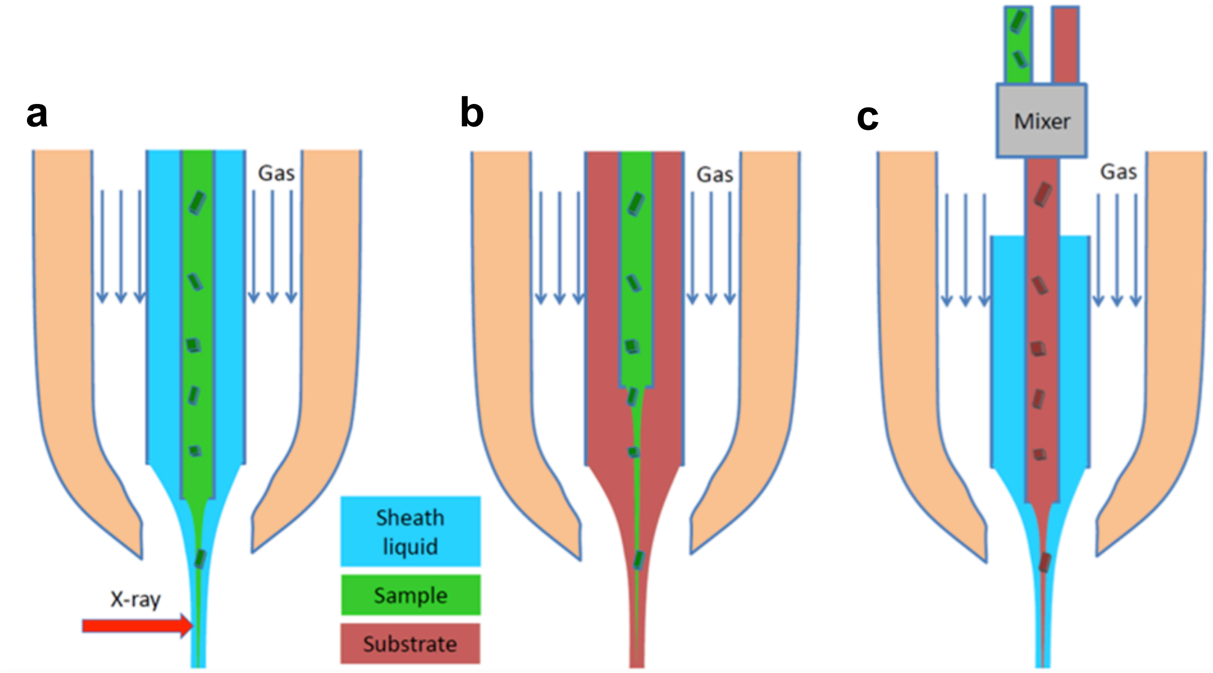

Supplementary Figure 9 A DFF injector, **a**, can be converted to a mix-and-inject apparatus for time-resolved crystallography **b** (as reported by Wang et al.) by retracting the sample line (green) and running the substrate in the sheath. **c** shows a more advanced set-up for longer time delays, obtained by combining a microfluidic mixer for rapid mixing of substrate and enzyme crystals with a DFF injector that is supplied with a separate sheath liquid.

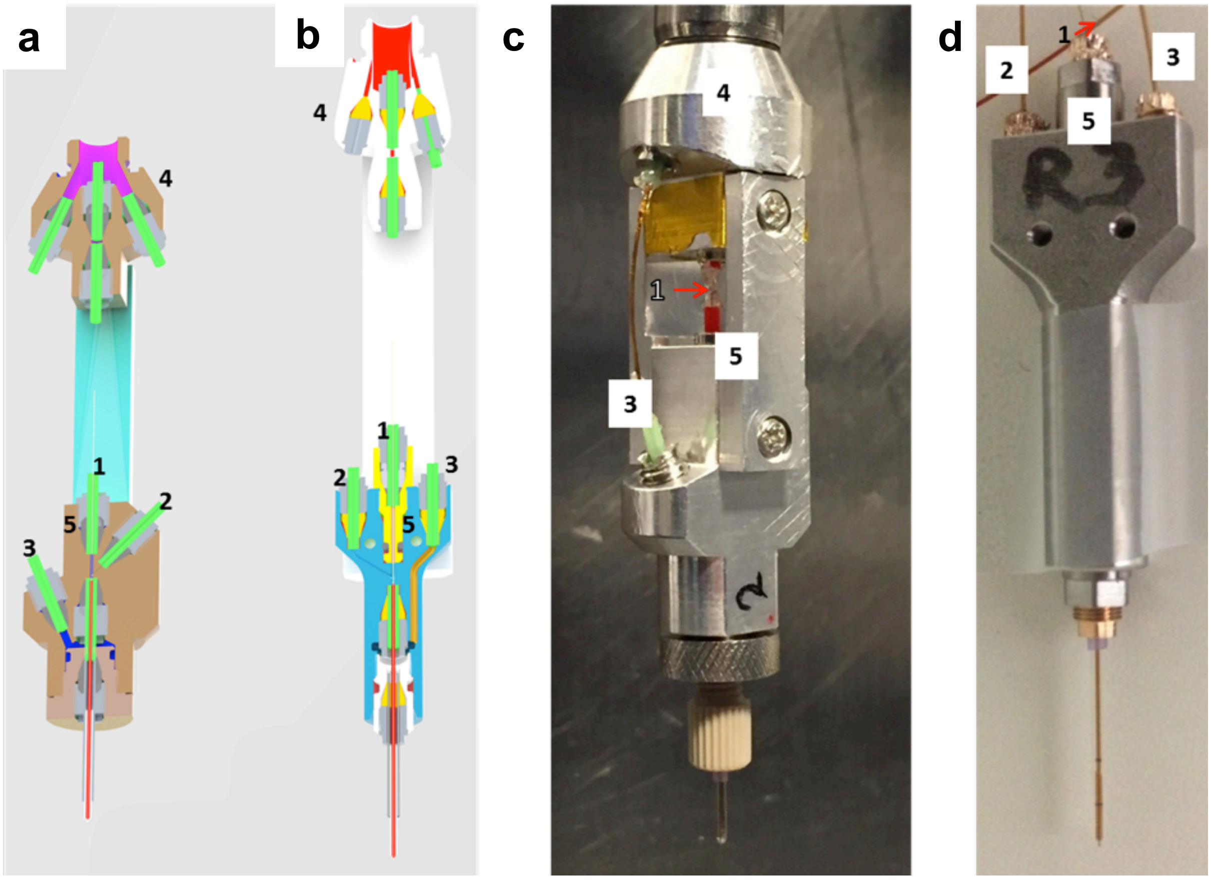

Supplementary Figure 10 Comparison of DFFN generations. **a** CAD-drawing of the first DFF injector generation. **b,** CAD-drawing of the second generation. **c**, assembled first generation DFF injector. These were machined from aluminum or bronze whereas the second generation, **d**, was produced by 3D metal sintering of steel. All injectors consist of inner line (1) for sample, sheath liquid line (2) and focusing gas line (3). They are mounted to the standard "nozzle rod" at the CXI endstation using an adaptor piece (4). The second generation can be operated without this adaptor piece when mounted to other instrumentation such as a Smargon goniometer. The position of the inner capillary relative to the gas orifice of the nozzle and the end of the sheath-liquid capillary can be adjusted by a screw (5). This screw was improved in the second generation and now allows re-purposing of the DFF injector to a mix-and-inject device.

Supplementary Table 1

	CpGV (5MND)	RNA pol II (5U5Q)	*Re*MBH	Dioxygenase (5TRX)
**Data collection**				
Space group	I23	$C222_1$	$P2_12_12_1$	$P2_12_12$
Cell dimensions				
a, *b*, *c* (Å)	103.4	223.4, 396.8, 287.9	74.6, 97.17, 122.0	111.6 154.9 101.8
α, β, γ (°)	90	90	90	90
Resolution (Å)	2.56-32.7 (2.56-2.65)[a]	3.80-40.6 (3.80-3.873)	2.20 – 38.61 (2.20-2.30)	2.38 - 34.5 (2.38 - 2.47)
R_{split}	2.72(15.31)	27.88(272.99)	26.61 (113.0)	37.5 (82.5)
SNR	35.21(8.57)	2.41 (0.48)	2.77 (0.96)	2.43 (3.42)
$CC_{1/2}$	0.999 (0.965)	0.960 (0.101)	0.912 (0.329)	0.811 (0.453)
CC^*	0.999 (0.991)	0.989 (0.428)	0.977 (0.704)	0.946 (0.790)
Completeness (%)	99.6(97.0)	99.0 (99.0)	99.9(99.9)	99.9 (99.5)
Redundancy	1755.5 (351.9)	54.9(13.0)	45.6(19.7)	26.5 (13.0)
Wilson B factor (Å^2)	23.5	140.9	49.7	35.9
**Refinement**				
Resolution (Å)	2.56-32.7	3.80-40.6		20 – 2.38
No. reflections	6052	123899		67578
R_{work} / R_{free}	0.154/0.207	0.22/0.27		0.18/0.23
No. atoms				
Protein	2042	31625		11621
Ligand/ion (specify/describe)	-	9		4 (Fe), 4 (Cl^-), 1 (Ca)
Water	80	-		439
B factors				
Protein	19.09	169.14		43.7
Ligand/ion	-	140.38		42.5 (Fe), 36.5 (Cl^-), 48.2 (Ca)
Water	17.35	-		40.4
R.m.s. deviations				
Bond lengths (Å)	0.003	0.003		0.011
Bond angles (°)	0.540	0.616		1.358
